# The Effect of Behavioral Couple Therapy on the Improvement of Mental Health and Reduction of Marital Conflict in Infertile Couples in Kermanshah: A Randomized Controlled Trial (RCT)

**Published:** 2019

**Authors:** Seyed Mojtaba Ahmadi, Jamile Shahverdi, Mansour Rezaei, Mitra Bakhtiari, Kheirollah Sadeghi, Fateme Veisy, Maryam Shahverdi

**Affiliations:** 1- Department of clinical psychology, School of Medicine, Shahid Beheshti University of Medical Sciences, Tehran, Iran; 2- Department of Psychology, Faculty of Medicine, Kermanshah University of Medical Sciences, Kermanshah, Iran; 3- Department of Biostatistics and Epidemiology, Social Development and Health Promotion Research Center, Kermanshah University of Medical Sciences, Kermanshah, Iran; 4- Department of Anatomy and Biology, Faculty of Medicine, Kermanshah University of Medical Science, Kermanshah, Iran; 5- Department of Clinical Psychology, School of Medicine, Iran University of Medical Sciences, Tehran, Iran; 6- Department of Information Technology, Payame Noor University Tehran West, Tehran, Iran

**Keywords:** Behavioral couple therapy, Infertility, Marital conflict, Mental health

## Abstract

**Background:**

Infertility is a common disorder, exposing couples to complication such as the loss of mental health and the increase of marital conflicts. The aim of this study was to evaluate the effect of behavioral couple therapy on the enhancement of mental health and reduction of marital conflicts.

**Methods:**

In this clinical trial, 24 couples were selected using convenience sampling and were divided randomly into control (12 couples) and experimental (12 couples) groups. Mental Health Questionnaire (GHQ-28) and Kansas Marital Conflict Scale (KMCS) were used to collect data. These questionnaires were filled and pretest, posttest and followup were done in two months. Data were analyzed by Repeated Measures Analysis of Variance, chi-square, independent sample T test, and Bonferroni tests using SPSS-16 software. The significant level of the test was 0.05.

**Results:**

The results of the data analysis between experimental and control groups of females in the marital conflict variable showed that the effect of time (p=0.002) and time and group interactional effect (p=0.001) were significant. Moreover, in both experimental and control groups of males, time effect was significant (p=0.01), but time and group interactional effect was not significant (p=0.14). Also, the results of the data analysis between experimental and control groups of females in the mental health and time effect was significant (p=0.001) and time and group interactional effect was significant as well (p=0.001). But in both experimental and control groups of males, time effect (p=0.71) and time and group interactional effect were not significant (p=0.60).

**Conclusion:**

Behavioral couple therapy can be used in the treatment of infertile couples, especially in women.

## Introduction

Infertility is defined as the absence of pregnancy following intercourse for one year without using contraceptives. According to researches conducted, 0.15 of couples have no children despite their tendency and 0.10 of them have less than the number of children they want to have (1). According to World Health Organization (WHO), 50 to 80 million people around the world are involved with primary or secondary infertility (2). Infertility prevalence in Iran has been reported to be 13.2%. The rate of primary infertility in Iran was 2.2 and secondary infertility was 3.2 (3).

The process of infertility can cause certain emotional aspects. There are cases which create pressure and emotional conflict which can be enhanced in time. Anxiety, depression and marital disruption are the results of pressure from infertility, lack of emotional and psychological support, and the stress of high costs of infertility treatment, the absence from work for treatment, the disappointing treatment outcome and the feeling of permanent infertility (4). The prevalence of psychiatric problems in infertile couples are estimated to be between 0.25 and 0.60 and the rate of depression and anxiety was significantly higher than the fertile group and the general population (5). One study in Bandar Abbas on 200 infertile women showed that 0.45% of them get higher scores than the cut-off point in the mental health questionnaire (6). In the meta-analysis conducted in Iran, prevalence of depression in infertile couples was 0.47%; the rate was 0.46 among women and 0.47% among men (7).

According to the findings of researches, high levels of anxiety and depression, decrease fertility even when IVF strategies are in process. According to Reed findings, anxiety and depression can cause failure and the repetition of treatment is required. 0.33% of infertile women suffer from depression while only 0.18% of fertile women face depression. Therefore, psychological problems associated with infertility are the most important challenge faced by infertile people which affect the their treatment (8). Infertility is one of the main causes of family relationships problem, disruption, divorce, lack of self-confidence and social isolation (6, 9). Studies have shown that infertile women compared with fertile women have less mental health and marital adjustment (10).

According to Saeedi et al., marital complain comes from the lack of coordination of needs and irresponsible behaviors among couples. Marital conflict when the response to individual differences is arising into a level of feeling of anger, hostility, in the couple’s relationship and become destructive (10).

Rice discussed two types of conflict in marriage in 1996, namely constructive conflict and destructive conflict. In constructive conflict, the focus is on problem solving, trust, and respect where there is little verbal contact and negative emotion between them. In destructive conflict, the couple attack each other instead of working on the problem and try to dominate the other, use negative expression to call each other and this causes a non–coherent relation between them (11).

Based on these issues, ignoring the psychological problems of infertility and just checking the medical aspect can be a major obstacle in the way in which the total understanding of infertility is shaped. Therefore, infertility must be seen through the realm of Behavioral Sciences at the same time. Obviously, referral of infertile couples to modern infertility treatment centers can induce the feeling that the health system only targets their physical problem and ignores their general conditions (12).

Despite the importance of conflicts in marital life, there have been very few researches on it yet. This research aimed to investigate the theory of couple behavioral treatment in social-cultural conditions in Iran with the goal of reducing the couple conflict and helping to increase their general health.

## Methods

This research is a randomized controlled clinical trial. Study population of this research consisted of all the infertile couples in Kermanshah who went to Motazedi infertility treatment center for the treatment of their infertility. The research sample consisted of 24 couples. Inclusion criteria included having a score higher than 22 in GHQ (General health questionnaire), the confirmation of the presence of general health problems based on the clinical interview structured for disorders DSM-IV-TR. Every person needed to consent to participate in the research by signing a consent form, non-interfering in the process of infertility treatment, confirming the absence of any other psychiatric disorder at the time of study and being married at least for two years. Exclusion criteria of the study included any significant disruption in the axis I, except those which are being studied, any physical disorder which can affect the process of psychotherapy, signs and symptoms of psychosis including hallucinations and delusions, exacerbation of depressive symptoms during treatment, and personality disorder on axis II. The evaluation of I, II axes was done by using structured interviews (SCID).

SCID is a semi-structured clinical interview that provides diagnosis based on DSM-IV. It includes two versions of SCID-I for Axis I disorder and SCID-II that examines personality disorders. In this study, SCID-I was used for the diagnosis of major depression. This tool was studied on 299 people who referred to psychiatric centers in Tehran, Iran. The results showed that the Persian version of SCID is a reliable tool for diagnosis for clinical research and educational purposes (13).

### Tools:

Two questionnaires were used in this research, namely general health questionnaire (GHQ-28) and Kansas Marital Conflict Scale (KMCS).

General health questionnaire (GHQ-28) was designed by Goldberg and Hiller. It contains 28 items and 4 subscales: 1) physical symptoms, 2) anxiety and insomnia, 3) social dysfunctions, and 4) depression. The scores lower than 22 on this questionnaire is a sign of good general health. By Cronbach’s alpha coefficients, reliability coefficient for the total scale has been reported 0.88 and for sub-scales from 0.66 to 0.85. Behjati ardekani reported the reliability of the questionnaire 0.85 based on test-retest method and 0.76 on by Cronbach’s coefficient (14).

Kansas Marital Conflict Scale (KMCS) is a test including 27 items that aims to measure the couple conflict. It has a good internal consistency. The alpha range for men comes to 0.91 to 0.95. Consistency of test-retest reliability in all 3 steps ranged between 0.64 to 0.96 (15). By Cronbach's alpha coefficients, reliability coefficient for the total scale has been reported 0.75.

### Statistical analysis:

Data were analyzed using SPSS-16 and descriptive statistics (The mean and standard deviation) and inferential statistics (Independent sample T test, mixed analysis of variance-between and within groups, repeated measures variance analysis and Bonferroni tests). The significant level of the test was 0.05.

### The research process:

First, a pretest was given to the infertile couples who had come to The Motazedi infertility treatment center which led to selecting 24 couples gaining at least 22 in general health. Then, they were divided randomly into two groups of test and control. While the control group received only the routine infertility treatment, the test group was subjected to intervention trial in 10 sessions of 90 *min* duration (Running one session every week). A posttest was done in both groups after the 10 session intervention trial. Follow up was done after 2 months. The summary of treatment meetings is reported here; the first session: stating the objectives and rules in the group, outlining the general principles of behavioral relation based treatment, and the concept of marital conflict. The second session: learning the basic principles of behavior therapy, and listening skills. The third session: learning to understand the generality of communication and expressing important principles in communication. The fourth session: understanding the concept of documents, types of marital attribution. The fifth session: teaching appropriate nonverbal and verbal behaviors and male–female communication differences. The sixth session: training the basic principles of reinforcement-punishment and mutual relations. The seventh session: learning of cognitive factors, and cognitive errors by couples. The eighth session: learning behavioral–communication modeling (six aspects). The ninth session: learning problem-solving skills. And the tenth session: conclusion, getting feedback and running posttest (15, 16).

This study has the certificate no.4451 by Kermanshah University of Medical Sciences Ethics Committee and has been registered in center of clinical trial of Iran under the no. IRCT 2014 010716121N1.

## Results

Half of the participants were females and the other half were males. 43.8% of them were educated (Graduated from university) and 45.8% of them were housewives, 35.4% were self-employed and 18.8% were clerks. Based on their economic situations (Reported by couples), they were mostly middle class families. They were married for 2 to 8 years. Primary infertility was observed in four couples. The results showed that there was no significant difference in demographic characteristics between the two groups.

The mean and the standard deviation of marital conflict and mental health in women and men in both control and experimental groups were assessed at three different time periods (Table 1).

**Table 1.** The mean and the standard deviation of marital conflict and mental health in women and men in both control and experimental groups, assessed at three different time periods according to sex

**Variables**	**Sex**	**Groups**	**Pretest**		**p-value**	**Posttest**		**p-value**	**Follow up**		**p-value**
			**Mean**	**SD**		**Mean**	**SD**		**Mean**	**SD**	
**Marital conflict**											
	Female	Experiment Control	75.50	7.47	0.17	88.75	7.93	0.006	90.33	6.05	0.001
			79.91	8.08		77.33	10.48		79.25	8.30	
	Male	Experiment Control	80.91	11.68	0.47	91.54	9.78	0.116	95.22	8.87	0.07
			84.25	11.20		85.41	9.61		88.08	10.27	
**Mental health**											
	Female	Experiment Control	61.75	7.91	0.49	41.58	10.21	p<0.001	43.08	10.80	0.001
			58.91	11.88		63.16	12.95		61.25	12.01	
	Male	Experiment Control	46.72	7.21	0.67	42.18	7.89	0.15	38.90	4.06	0.02
			45.08	9.04		46.50	8.69		45.25	7.87	

In the marital conflict scale, among both males and females in experimental groups, a rise in scores in posttest and follow up compared to pre-test was seen. In Kansas Marital Conflict Scale (KMCS), marital conflict decreases with the increase in scores. However, there was no significant variety in scores in control groups. In the scale of mental health, both females and males in experimental group had a remarkable loss of scores in posttest and follow up (the lower the mental health questionnaire score, the better the mental health). However, there is a rise in scores among members of control group (Table 1).

Repeated Measures Analysis of Variance in marital conflict and mental health variables showed that (p<0.05) the changes in periods of time, method of therapy, interactive effects of time and group can be significant among females. However, interactive effect of time and group is not significant among males (p=0.60) (Table 2).

**Table 2.** Within-subject effects of marital conflicts and mental health according to sex

**Variables**	**Sex**	**Variables**	**SS**	**DF**	**MS**	**F**	**p**
**Marital conflict**							
	Female	time	602.083	1	601.083	12.710	0.002
		time*group	720.750	1	720.750	15.21	0.001
	Male	time	981.021	1	981.021	6.745	0.016
		time*group	325.521	1	325.521	2.238	0.149
**Mental health**							
	Female	time	800.333	1	800.333	15.092	0.001
		time*group	1323.000	1	1323.000	24.948	0.001
	Male	time	157.688	1	157.688	3.609	0.71
		time*group	172.521	1	172.521	3.948	0.60

### Marital conflict among females:

The results of analyzing data among females showed that the effect of time (p=0.002) and time and group interactional effect were significant (p=0.001). Considering the significant effect of time and group, analysis of variance of Repeated Measures Analysis of Variance was performed in experimental and control groups separately, and the comparison between pretest, posttest and follow up results was done between intervention and control groups.

For Repeated Measures Analysis of Variance, first, Mauchly’s test was analyzed. The homogeneity of the variance covariance matrix exists in experimental group (p=0.73) and in control group (p=0.81). The results showed that marital conflict did not show a significant change during the time in the control group (p=0.47), but in the experiment group, there was a significant increase during the time (p<0.001).

Bonferroni's results showed that the average change of scores of marital conflict in the post-test compared with the pretest increased about 13.25 scores (p=0.003) and in follow up test compared with the pretest increased about 14.83 scores (p=0.001). These changes were significant; however, there was a reduction in follow up phase compared with the pretest of 1.58 scores that is not significant.

**Figure 1.**
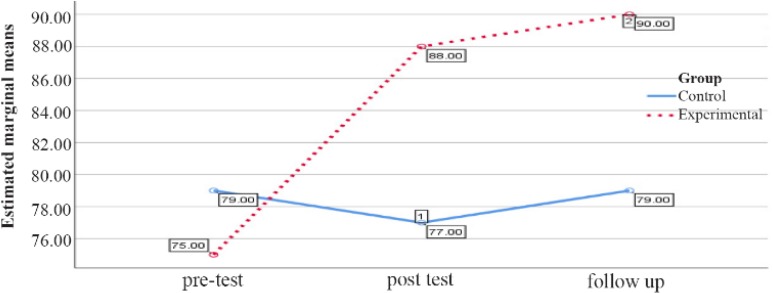
Graph of changes in marital conflict scores between female experimental and control groups

The comparison between the groups regarding marital conflict variable in the female groups in the pretest, posttest and follow up stages is reported in table 1. As shown in table 1, the marital conflict in the pretest stage does not show a significant difference (p=0.17), but in the posttest phase, an increase in the experimental group compared with the control group (p=0.006) was observed. In the follow up phase, this increase in marital conflict continued to be higher than the control group (p=0.001) (Table 1, Figure 1).

### Marital conflict among males:

The results of analyzing data among males showed that the effect of time was significant (p=0.01), but time and group interactional effect was not significant (p=2.23). To analyze the trend of changes in posttest and follow up, Repeated Measures Analysis of Variance was performed separately.

For Repeated Measures Analysis of Variance, first, Mauchly’s test was analyzed. The homogeneity of the variance covariance matrix exists in experimental group (p=0.20) and in control group (p=0.09). The results showed that marital conflict did not show a significant change during the time in the control group (p=0.62), but in the experiment group, there was a significant change during the time (p=0.001).

Bonferroni's results showed that the average change of scores of marital conflict in the post-test compared with the pretest increased about 10.63 scores (p=0.07) which is not significant, but in follow up test compared with the pretest, it increased about 14.31 scores (p=0.04); this change was significant.

**Figure 2.**
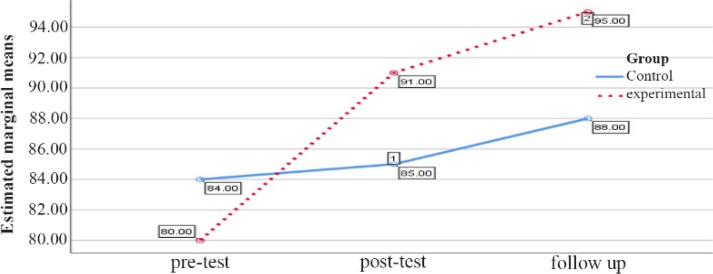
Graph of changes in marital conflict scores between male experimental and control groups

The comparison between the groups regarding the marital conflict variable in the male group in the pretest, posttest and follow up stages is reported in table 1. As shown in table 1, the marital conflict in the pretest stage does not show a significant difference (p=0.47), and in the posttest and follow up stage, no significant difference between the control group and the experimental group (p>0.05) is observed (Table 1, Figure 2).

### Mental health among females:

The results of analyzing data among females showed that the effect of time (p=0.001) and time and group interactional effect were significant (p=0.001). Considering the significant effect of time and group, Repeated Measures Analysis of Variance was performed in experimental and control groups, and the comparison between pretest, posttest and follow up results was done between the intervention and control groups.

For Repeated Measures Analysis of Variance, first, Mauchly’s test was analyzed. The homogeneity of the variance covariance matrix exists in experimental group (p=0.554) and in control group (p=0.002). The results showed that mental health did not show a significant change during the time in the control group (p=2.48), but in the experiment group, there was a significant increase during the time (p<0.001).

Bonferroni's results showed that the average change of scores of mental health in the posttest compared with the pretest increased about 20.17 scores (p=0.003) and in follow up test compared with the pretest decreased about 1.5 scores (P=1). The score was not significant (p=1).

**Figure 3.**
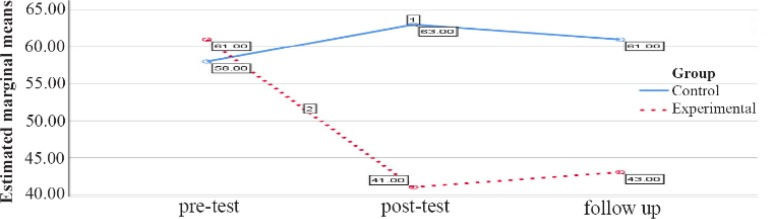
Graph of changes in mental health score between female experimental and control groups

The comparison between the groups regarding the mental health variable in the female group in the pretest, posttest and follow up stages is reported in table 1. As shown in table 1, the pretest stage score does not show a significant difference (p=0.49), but in the posttest phase, a decrease in the mean of the experimental group compared with the control group (p<0.001) was observed. In the follow up phase, this decrease in mental health continued to be higher than control group (p=0.001) (Table 1, Figure 3).

### Mental health among males:

The results of analyzing data among males showed that the effect of time (p=0.71) and time and group interactional effect were not significant (p=0.60, f=3.94). Considering the significant effect of time and group, Repeated Measures Analysis of Variance was performed in experimental and control groups, and the comparison between pretest, posttest and follow up results was done between the intervention and control groups.

**Figure 4.**
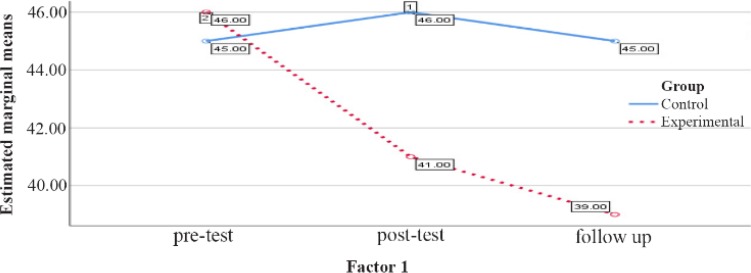
Graph of changes in mental health score between male experimental and control groups

The comparison between the groups regarding the mental health variable in the male group in the pretest, posttest and follow up stages is reported in table 1. As shown in table 1, pretest stage score does not show a significant difference (p=0.49), but in the posttest phase, a decrease in the mean of experimental group compared with the control group (p<0.001) was observed which is not significant. In the follow up phase, this decrease in mental health continued to be higher than control group (p=0.001) (Table 1, Figure 4).

## Discussion

Infertility has devastating psycho-social consequences on infertile couples. It causes a worldwide challenge (17). Infertility usually causes numerous emotional reactions like depression, anxiety, and stress (18). Mental health means the absence of disease in individuals. It is one of the most important factors in improving human life. The World Health Organization (WHO) defines mental health as the total ability to play social, emotional and physical roles. Family has a very important role in mental health, because, our first experience of living begins in the family (19).

Goldstein defines mental health as the balance between the members and the environment to achieve self-actualization. In fact, mental health is something more than being healthy. People with proper mental health encounter less confusion and conflict in marital life. Regardless of the source of a breach in the relationship, learning communicative skills can help couples in development of appropriate communicative behaviors and decreasing marital conflict (20). This research aimed to show the effect of behavioral couple therapy on increasing mental health and decreasing marital conflict among infertile couples. The results of the covariance analysis showed that there is a significant difference between the experimental group who received the behavioral couple therapy and the control group who had no treatment or therapy. In this regard, the findings of this research are in line with the results of Frederiksen et al. (20), Mir Ahmadi et al. (21), Abdulqader (22), Khalatbari et al. (23), Soltani et al. (24), Soleimani et al. (17), and Hasani (25).

Frederiksen et al. showed in their research that psychosocial interventions for couples in treatment for infertility, in particular CBT, could be efficacious, both in reducing psychological distress and in improving clinical pregnancy rates (20). In a study by Mir Ahmadi et al., the results showed that short term couple therapy is effective for increasing mental health and decreasing depression (21).

Khalatbari et al. in their study showed that the behavioral cognitive therapy has a significant positive influence over depression of the experimental group compared with the control group which had received no therapeutic interference (23). Soltani et al. showed that emotionally focused therapy could reduce the rate of depression, anxiety and stress in infertile couples, regardless of the gender, namely male or female, as the cause of infertility (24).

Soleimani et al. showed in their research that EFT-C significantly affected marital adjustment and sexual satisfaction (17). Hasani in his study on “Comparing the effectiveness of behavioral-cognitive and emotional output in an emotion-focused depression in infertile husbands” showed that this treatment had some effects on the infertile males treatment (25).

The results of these researches showed that the rate of marital problems was high among infertile couples before the interventions but it was meaningfully reduced after their treatment. The scores were higher in females in experimental group in comparison with the control group. But it didn’t have any significant difference in males in both experimental and control groups.

Abdulqader in his research showed that marital satisfaction after psychological intervention increased among infertile couples (22). Concerning the marital conflicts, the results of this research were in alignment with researches mentioned before. Soudani et al. researched the effectiveness of Bernstein behavioral-association therapy on marital therapy. The results showed that it can decrease marital conflicts (16).

Nazari et al. studied the effectiveness of fortified cognitive-behavioral and blended couple therapy on increasing the couple satisfaction. The results showed that both therapies had good effects on couples’ satisfaction. However, fortified cognitive-behavioral therapy showed to be more effective than the blended couples therapy (26).

Behavioral couple therapy was helpful to reduce marital conflict and improved mental health of infertile couples after following the therapy sessions in two months. Perhaps as the main cause of infertility, the couple's conflicts and psychological issues can affect even the physical infertility treatment. Moreover, there were two pregnancies in experimental group after the intervention. These results show that for those infertile couples who go to the gynecologist for treatment without any significant physical cause for infertility, counseling and psychotherapy centers are the best options and behavioral couple therapy which can be very effective as part of the treatment process should be used for them. The historical value of the research showed that educational interventions in the field of couple therapy can increase the mental health and reduce the marital conflicts among couples. Using this treatment plan can help to increase couples’ mental health, leading to the overall improvement in relations of couples.

## Conclusion

According to the results of the present study, it can be concluded that the behavioral couple therapy plays an important role in mental health and marital conflict between males and females. And according to the results, the benefit of this method is more for females than males. It is recommended that infertile couples who go to the gynecologist without any physical cause for infertility, be sent to counseling centers as part of the treatment process. Educational interventions in the field of couple’s therapy could increase the general health and reduce the marital conflicts. Generalization of the results requires further investigation, but behavioral couple therapy has an important effect on the infertile couples and helps them in the procedure of infertility treatment.
